# SARS-CoV-2 Molecular Evolutionary Dynamics in the Greater Accra Region, Ghana

**DOI:** 10.3201/eid2904.221410

**Published:** 2023-04

**Authors:** Bright Adu, Joseph H.K. Bonney, Beverly Egyir, Isaac Darko Otchere, Prince Asare, Francis E. Dennis, Evelyn Yayra Bonney, Richard Akuffo, Ivy A. Asante, Evangeline Obodai, Selassie Kumordjie, Joyce Appiah-Kubi, Quaneeta Mohktar, Hilda Opoku Frempong, Franklin Asiedu-Bekoe, Mildred A. Adusei-Poku, James O. Aboagye, Bright Agbodzi, Clara Yeboah, Seyram B. Agbenyo, Peace O. Uche, Keren O. Attiku, Bernice Twenewaa Sekyere, Dennis Laryea, Kwame Buabeng, Helena Lamptey, Anita Ghansah, Dorothy Yeboah-Manu, Abraham K. Anang, William K. Ampofo, George B. Kyei, John K. Odoom

**Affiliations:** Noguchi Memorial Institute for Medical Research, University of Ghana College of Health Sciences, Legon, Ghana (B. Adu, J.H.K. Bonney, B. Egyir, I.D. Otchere, P. Asare, F.E. Dennis, E.Y. Bonney, R. Akuffo, I.A. Asante, E. Obodai, S. Kumordjie, J. Appiah-Kubi, Q. Mohktar, H. Opoku Frempong, J.O. Aboagye, B. Agbodzi, C. Yeboah, S.B. Agbenyo, P.O. Uche, K.O. Attiku, B. Twenewaa Sekyere, D. Laryea, K. Buabeng, H. Lamptey, A. Ghansah, D. Yeboah-Manu, A.K. Anang, W.K. Ampofo, G.B. Kyei, J.K. Odoom);; Ghana Health Service, Accra, Ghana (F. Asiedu-Bekoe);; University of Ghana Medical School, Accra (M.A. Adusei-Poku);; University of Ghana Medical Centre, Legon (G.B. Kyei)

**Keywords:** COVID-19, 2019 novel coronavirus disease, coronavirus disease, severe acute respiratory syndrome coronavirus 2, SARS-CoV-2, viruses, respiratory infections, zoonoses, variants, genomic surveillance, Accra Metropolitan District, Greater Accra Region, Ghana

## Abstract

To assess dynamics of SARS-CoV-2 in Greater Accra Region, Ghana, we analyzed SARS-CoV-2 genomic sequences from persons in the community and returning from international travel. The Accra Metropolitan District was a major origin of virus spread to other districts and should be a primary focus for interventions against future infectious disease outbreaks.

 The emergence of SARS-CoV-2 variants with superior transmissibility or immune evasion advantages may cause outbreaks and dominate transmission in a population (*1*). Thus, keeping track of the dynamics of variant transmissions in a population is crucial for developing timely and appropriate responses to outbreaks.

In Ghana, whereas the entire population experienced the COVID-19 pandemic, most infections were primarily recorded in the Greater Accra Region (GAR), the most densely populated region in Ghana with the smallest landmass (*2*). The genetic diversity of SARS-CoV-2 infections in Ghana during early (*3*) and recent (*4*) transmissions showed initial transmission driven by multiple lineages of the virus, after which the Alpha, Delta, and Omicron variants dominated. To gain information about the dynamics of SARS-CoV-2 spread within the GAR, the epicenter of the COVID-19 outbreak in Ghana, we performed a detailed analysis of variants.

We analyzed 1,163 SARS-CoV-2 genomic sequences from 834 community samples collected from 14 of the 21 districts in the GAR and 329 from returning international travelers (Table) during March 2020–February 2022. We extracted RNA from oro/nasopharyngeal swab samples of patients by using a QIAamp Viral RNA Mini Kit (QIAGEN, https://www.qiagen.com).

**Table:** Distribution of SARS-CoV-2 sequences analyzed by district of Ghana and origin of international travelers

Origin of travelers	Sequences, no. (%)
Ghana, n = 834	
Accra Metropolitan District	421 (50.5)
Ashaiman Municipal	1 (0.1)
Adenta Municipal	41 (4.9)
Ga East	19 (2.3)
Ga Central	8 (1.0)
Ga South	6 (0.7)
Ga West	21 (2.5)
Kpone Katamanso	1 (0.1)
La-Dade Kotopon	21 (2.5)
La-Nkwantanang Madina	9 (1.1)
Ledzokuku Krowor	6 (0.7)
Ningo Prampram	1 (0.1)
Shai Osudoku	12 (1.4)
Tema Municipal	25 (3.0)
Unnamed district*	242 (29.0)
World, n = 329	
Africa	159 (48.3)
Asia	85 (25.8)
Europe	57 (17.3)
North America	28 (8.5)

*Samples from within the Greater Accra Region but with no clear indication of the specific district.

We prepared complementary DNA by using the LunaScript RT Super Mix Kit (New England BioLabs, https://www.neb.com). For amplicon generation, we used either the ARTIC nCoV-2019 version 3 primers (Artic Network, https://artic.network) (batch 1 samples, collected before July 2021) or the Midnight RT PCR Expansion kit (Oxford Nanopore Technologies, https://www.nanoporetech.com) (batch 2 samples, collected after July 2021). We sequenced batch 1 samples on Illumina MiSeq after library preparation with an Illumina DNA prep kit (https://www.illumina.com) and batch 2 samples on GridION after library preparation with SQK-RBK110.96 kit (Oxford Nanopore Technologies).

For both batches of samples, we analyzed reads by using the ARTIC version 1.2 field bioinformatics pipeline (https://github.com/artic-network/fieldbioinformatics). We assigned Lineages by using Pangolin version 4.1.3 with pangolin-data version 1.17 (*5*).

For the phylogenetic analysis, we first aligned sequences in MAFFT version 7.490 (*6*). We inferred the maximum-likelihood tree topology of the variable positions with 1,000 bootstraps by using IQ-TREE version 2.0.7 (*7*) with the general time reversible nucleotide substitution model. We populated the maximum-likelihood tree with sampling dates by using TreeTime version 0.8.6 (*8*) and assuming a mean constant nucleotide substitutions per site per year rate of 8.0 × 10^−4^ (*9*) after excluding outlier sequences. We then rerooted the final dated tree with 936 sequences to the initial wild-type SARS-CoV-2 strain (GenBank accession no. NC_045512.2) and visualized in R version 4.1.2 (https://www.r-project.org) by using ggtree version 3.2.1 and ggtreeExtra version 1.4.2 packages (*10*). For the import–export analysis, we labeled the internal nodes and external leaves of the dated phylogeny with the location/district of sample origin by using TreeTime. We inferred the number of state changes from one location/district to another and time of event by using a python script developed by Wilkinson Lab (https://github.com/CERI-KRISP/africa-covid19-genomics/tree/main/python_scripts).

Of the 152,896 SARS-CoV-2 infections reported in Ghana by February 28, 2022, the GAR alone contributed 90,267 (59.04%) (Appendix Table 1). Of the 21 districts in the GAR, the Accra Metropolitan District (AMD) consistently contributed ≈50% of reported SARS-CoV-2 infections in the region since the outbreak began in Ghana (https://ghs.gov.gh/covid19/archive.php). This finding mirrors our finding of 50.5% of sequences from the region being from the AMD (Table). Although all analyzed sequences (Appendix Table 2) came from the GAR, representative metadata for some samples were not indicated by all districts. Those districts were grouped as “Unnamed District” and accounted for 29% of the sequences, most of which were the Alpha variant (Appendix Figure 1).

Because different lineages have dominated SARS-CoV-2 transmission in Ghana at different periods, we categorized the data into the main SARS-CoV-2 variants (Alpha, Beta, Delta, Eta, Omicron, and others). From the phylogenetic analysis, the SARS-CoV-2 variants circulating in the districts of the GAR and those from returning international travelers resolved into 5 major clusters corresponding to defined categories (Appendix Figure 2, panel A). Sequences from the returning international travelers colocalized with the GAR samples, suggesting minimal divergence. We found that an estimated 77 SARS-CoV-2 variant introduction events occurred in the AMD, mainly from other parts of Africa and other districts (Appendix Figure 2, panel B). In contrast, there were an estimated 185 SARS-CoV-2 variant exportation events from the AMD, mainly to the other districts of the GAR and to relatively fewer to countries outside Ghana (Appendix Figure 2, panels C, D). Of those variant exportation events, 153 were to other districts in the GAR, making the AMD a prime district for targeted interventions aimed at reducing the spread of SARS-CoV-2 and other infectious pathogens.

In conclusion, SARS-CoV-2 genomic surveillance in the GAR of Ghana revealed the pattern of spread of variants among districts of the region, demonstrating the role of the AMD in the spread of SARS-CoV-2 in the GAR. We propose that the AMD should be a primary focus in public health interventions aimed at controlling SARS-CoV-2 and other future infectious disease outbreaks in the GAR.

Appendix Additional information for study of SARS-CoV-2 molecular evolutionary dynamics in the Greater Accra Region of Ghana.
