# Supplementary material for: SARS-CoV-2 Molecular Evolutionary Dynamics in the Greater Accra Region, Ghana
Source: Emerg Infect Dis. 2023 Apr;29(4):862–5. doi: 10.3201/eid2904.221410 (PMC10045712; doi:10.3201/eid2904.221410)
Supplement: Appendix — Additional information for study of SARS-CoV-2 molecular evolutionary dynamics in the Greater Accra Region of Ghana. [file 22-1410-Techapp-s1.pdf]

Article DOI: https://doi.org/10.3201/eid2904.221410

EID cannot ensure accessibility for supplementary materials supplied by authors. Readers who have difficulty accessing supplementary content should contact the authors for assistance.

SARS-CoV-2 Molecular Evolutionary Dynamics in the Greater Accra Region, Ghana

Appendix

Appendix Table 1. SARS-CoV-2 infections in Ghana by region from March 2020 to February 28, 2022*

Region	SARS-CoV-2 infections (count)	Percentage (%)
Greater Accra	90,267	59.04
Ashanti	22,292	14.58
Western	8,311	5.44
Eastern	7,032	4.60
Volta	5,954	3.89
Central	5,402	3.53
Bono East	2,966	1.94
Bono	2,332	1.53
Northern	1,863	1.22
Upper East	1,733	1.13
Ahafo	1,135	0.74
Western North	1,109	0.73
Oti	930	0.61
Upper West	895	0.59
North East	384	0.25
Savannah	291	0.19
Total	152,896	100.00

*Data source. Ghana Health Service COVID-19 outbreak response management updates. (https://www.ghs.gov.gh/covid19/archive.php).

Appendix Table 2. GISAID accession numbers of SARS-CoV-2 genomic sequences used in the study

gisaid_epi_isl	gisaid_epi_isl	gisaid_epi_isl	gisaid_epi_isl	gisaid_epi_isl	gisaid_epi_isl	gisaid_epi_isl
Samples from the Greater Accra region						
EPI_ISL_422382	EPI_ISL_2508389	EPI_ISL_8065554	EPI_ISL_8065656	EPI_ISL_8065756	EPI_ISL_8065853	EPI_ISL_8065956
EPI_ISL_422384	EPI_ISL_2508390	EPI_ISL_8065555	EPI_ISL_8065655	EPI_ISL_8065757	EPI_ISL_8065856	EPI_ISL_8065958
EPI_ISL_422404	EPI_ISL_2508391	EPI_ISL_8065556	EPI_ISL_8065654	EPI_ISL_8065758	EPI_ISL_8065858	EPI_ISL_8065959
EPI_ISL_422387	EPI_ISL_2508392	EPI_ISL_8065562	EPI_ISL_8065657	EPI_ISL_8065750	EPI_ISL_8065859	EPI_ISL_8065960
EPI_ISL_422405	EPI_ISL_2508393	EPI_ISL_8065558	EPI_ISL_8065658	EPI_ISL_8065752	EPI_ISL_8065857	EPI_ISL_8065961
EPI_ISL_422390	EPI_ISL_2508394	EPI_ISL_8065559	EPI_ISL_8065659	EPI_ISL_8065761	EPI_ISL_8065862	EPI_ISL_8065962
EPI_ISL_422394	EPI_ISL_2508395	EPI_ISL_8065560	EPI_ISL_8065662	EPI_ISL_8065763	EPI_ISL_8065863	EPI_ISL_8065964
EPI_ISL_515083	EPI_ISL_2508396	EPI_ISL_8065561	EPI_ISL_8065664	EPI_ISL_8065768	EPI_ISL_8065865	EPI_ISL_8065963
EPI_ISL_515084	EPI_ISL_8065519	EPI_ISL_8065557	EPI_ISL_8065663	EPI_ISL_8065759	EPI_ISL_8065861	EPI_ISL_8065965
EPI_ISL_515085	EPI_ISL_8065520	EPI_ISL_8065563	EPI_ISL_8065661	EPI_ISL_8065762	EPI_ISL_8065864	EPI_ISL_8065966
EPI_ISL_515082	EPI_ISL_8065521	EPI_ISL_8065564	EPI_ISL_8065665	EPI_ISL_8065764	EPI_ISL_8065860	EPI_ISL_8065967
EPI_ISL_422397	EPI_ISL_8065524	EPI_ISL_8065565	EPI_ISL_8065666	EPI_ISL_8065765	EPI_ISL_8065866	EPI_ISL_8065970
EPI_ISL_422406	EPI_ISL_8065522	EPI_ISL_8065570	EPI_ISL_8065668	EPI_ISL_8065766	EPI_ISL_8065869	EPI_ISL_8065969
EPI_ISL_422398	EPI_ISL_8065517	EPI_ISL_8065566	EPI_ISL_8065669	EPI_ISL_8065767	EPI_ISL_8065870	EPI_ISL_8065968
EPI_ISL_422402	EPI_ISL_8065518	EPI_ISL_8065567	EPI_ISL_8065660	EPI_ISL_8065760	EPI_ISL_8065873	EPI_ISL_8065972
EPI_ISL_422403	EPI_ISL_8065523	EPI_ISL_8065568	EPI_ISL_8065667	EPI_ISL_8065769	EPI_ISL_8065867	EPI_ISL_8065971
EPI_ISL_422400	EPI_ISL_2361908	EPI_ISL_8065569	EPI_ISL_8065670	EPI_ISL_8065790	EPI_ISL_8065868	EPI_ISL_8065975
EPI_ISL_422399	EPI_ISL_2361909	EPI_ISL_8065572	EPI_ISL_8065671	EPI_ISL_8065773	EPI_ISL_8065872	EPI_ISL_8065974
EPI_ISL_422401	EPI_ISL_2376383	EPI_ISL_8065571	EPI_ISL_8065672	EPI_ISL_8065785	EPI_ISL_8065871	EPI_ISL_8065973
EPI_ISL_515103	EPI_ISL_2361910	EPI_ISL_8065577	EPI_ISL_8065673	EPI_ISL_8065786	EPI_ISL_8065876	EPI_ISL_8065977
EPI_ISL_515098	EPI_ISL_2508381	EPI_ISL_8065575	EPI_ISL_8065674	EPI_ISL_8065789	EPI_ISL_8065874	EPI_ISL_8065976
EPI_ISL_515100	EPI_ISL_2508382	EPI_ISL_8065573	EPI_ISL_8065675	EPI_ISL_8065795	EPI_ISL_8065877	EPI_ISL_8065978
EPI_ISL_515099	EPI_ISL_2508380	EPI_ISL_8065576	EPI_ISL_8065692	EPI_ISL_8065796	EPI_ISL_8065878	EPI_ISL_8065979
EPI_ISL_515101	EPI_ISL_2508383	EPI_ISL_8065574	EPI_ISL_8065702	EPI_ISL_8065779	EPI_ISL_8065875	EPI_ISL_8065980
EPI_ISL_515086	EPI_ISL_2508384	EPI_ISL_8065578	EPI_ISL_8065680	EPI_ISL_8065771	EPI_ISL_8065880	EPI_ISL_8065982
EPI_ISL_515087	EPI_ISL_2361911	EPI_ISL_8065580	EPI_ISL_8065691	EPI_ISL_8065772	EPI_ISL_8065879	EPI_ISL_8065981
EPI_ISL_515089	EPI_ISL_2361912	EPI_ISL_8065579	EPI_ISL_8065697	EPI_ISL_8065774	EPI_ISL_8065883	EPI_ISL_8065983
EPI_ISL_515088	EPI_ISL_2361913	EPI_ISL_8065581	EPI_ISL_8065698	EPI_ISL_8065775	EPI_ISL_8065881	EPI_ISL_8065984
EPI_ISL_515090	EPI_ISL_2376384	EPI_ISL_8065583	EPI_ISL_8065686	EPI_ISL_8065776	EPI_ISL_8065882	EPI_ISL_8065985
EPI_ISL_515091	EPI_ISL_2376385	EPI_ISL_8065584	EPI_ISL_8065693	EPI_ISL_8065777	EPI_ISL_8065884	EPI_ISL_11765145
EPI_ISL_515093	EPI_ISL_2361914	EPI_ISL_8065585	EPI_ISL_8065676	EPI_ISL_8065778	EPI_ISL_8065885	EPI_ISL_11765152
EPI_ISL_515105	EPI_ISL_2376386	EPI_ISL_8065587	EPI_ISL_8065679	EPI_ISL_8065780	EPI_ISL_8065888	EPI_ISL_11765153
EPI_ISL_515111	EPI_ISL_2361915	EPI_ISL_8065588	EPI_ISL_8065681	EPI_ISL_8065781	EPI_ISL_8065890	EPI_ISL_11765154
EPI_ISL_515096	EPI_ISL_8065525	EPI_ISL_8065589	EPI_ISL_8065684	EPI_ISL_8065783	EPI_ISL_8065891	EPI_ISL_11765161
EPI_ISL_515109	EPI_ISL_2361904	EPI_ISL_8065590	EPI_ISL_8065685	EPI_ISL_8065784	EPI_ISL_8065887	EPI_ISL_11765174
EPI_ISL_515092	EPI_ISL_2348486	EPI_ISL_8065591	EPI_ISL_8065687	EPI_ISL_8065788	EPI_ISL_8065889	EPI_ISL_11765176
EPI_ISL_515094	EPI_ISL_2361905	EPI_ISL_8065592	EPI_ISL_8065688	EPI_ISL_8065791	EPI_ISL_8065886	EPI_ISL_11765177
EPI_ISL_515095	EPI_ISL_8065527	EPI_ISL_8065594	EPI_ISL_8065689	EPI_ISL_8065792	EPI_ISL_8065894	EPI_ISL_11765138
EPI_ISL_515106	EPI_ISL_8065526	EPI_ISL_8065595	EPI_ISL_8065690	EPI_ISL_8065797	EPI_ISL_8065895	EPI_ISL_11765149
EPI_ISL_515107	EPI_ISL_2376387	EPI_ISL_8065596	EPI_ISL_8065701	EPI_ISL_8065798	EPI_ISL_8065896	EPI_ISL_11765175
EPI_ISL_515102	EPI_ISL_2376388	EPI_ISL_8065597	EPI_ISL_8065682	EPI_ISL_8065799	EPI_ISL_8065892	EPI_ISL_11765151
EPI_ISL_515108	EPI_ISL_2361916	EPI_ISL_8065598	EPI_ISL_8065696	EPI_ISL_8065800	EPI_ISL_8065897	EPI_ISL_11765155
EPI_ISL_515110	EPI_ISL_2361906	EPI_ISL_8065599	EPI_ISL_8065677	EPI_ISL_8065770	EPI_ISL_8065893	EPI_ISL_11765157
EPI_ISL_515104	EPI_ISL_2361922	EPI_ISL_8065600	EPI_ISL_8065678	EPI_ISL_8065782	EPI_ISL_8065900	EPI_ISL_11765159
EPI_ISL_515112	EPI_ISL_2508386	EPI_ISL_8065601	EPI_ISL_8065683	EPI_ISL_8065787	EPI_ISL_8065903	EPI_ISL_11765160
EPI_ISL_515097	EPI_ISL_2361907	EPI_ISL_8065602	EPI_ISL_8065694	EPI_ISL_8065793	EPI_ISL_8065901	EPI_ISL_11765167
EPI_ISL_1018099	EPI_ISL_2361919	EPI_ISL_8065603	EPI_ISL_8065695	EPI_ISL_8065794	EPI_ISL_8065898	EPI_ISL_11765170

gisaid_epi_isl	gisaid_epi_isl	gisaid_epi_isl	gisaid_epi_isl	gisaid_epi_isl	gisaid_epi_isl	gisaid_epi_isl
EPI_ISL_1018100	EPI_ISL_8065528	EPI_ISL_8065604	EPI_ISL_8065699	EPI_ISL_8065802	EPI_ISL_8065899	EPI_ISL_11765171
EPI_ISL_1018101	EPI_ISL_8065529	EPI_ISL_8065605	EPI_ISL_8065700	EPI_ISL_8065803	EPI_ISL_8065902	EPI_ISL_11765173
EPI_ISL_1018089	EPI_ISL_2508373	EPI_ISL_8065606	EPI_ISL_8065708	EPI_ISL_8065801	EPI_ISL_8065904	EPI_ISL_11765143
EPI_ISL_1018088	EPI_ISL_8065531	EPI_ISL_8065607	EPI_ISL_8065715	EPI_ISL_8065804	EPI_ISL_8065908	EPI_ISL_11765142
EPI_ISL_1018090	EPI_ISL_8065530	EPI_ISL_8065608	EPI_ISL_8065710	EPI_ISL_8065805	EPI_ISL_8065905	EPI_ISL_11765140
EPI_ISL_1018091	EPI_ISL_2361918	EPI_ISL_8065609	EPI_ISL_8065713	EPI_ISL_8065806	EPI_ISL_8065906	EPI_ISL_11765162
EPI_ISL_1018092	EPI_ISL_2361917	EPI_ISL_8065610	EPI_ISL_8065714	EPI_ISL_8065807	EPI_ISL_8065907	EPI_ISL_11765150
EPI_ISL_1018093	EPI_ISL_8065532	EPI_ISL_8065611	EPI_ISL_8065704	EPI_ISL_8065817	EPI_ISL_8065910	EPI_ISL_11765156
EPI_ISL_1018095	EPI_ISL_8065533	EPI_ISL_8065612	EPI_ISL_8065705	EPI_ISL_8065809	EPI_ISL_8065909	EPI_ISL_11765144
EPI_ISL_1018094	EPI_ISL_2361924	EPI_ISL_8065613	EPI_ISL_8065706	EPI_ISL_8065813	EPI_ISL_8065912	EPI_ISL_11765168
EPI_ISL_1018096	EPI_ISL_8065534	EPI_ISL_8065614	EPI_ISL_8065707	EPI_ISL_8065823	EPI_ISL_8065915	EPI_ISL_11765166
EPI_ISL_1018097	EPI_ISL_2361923	EPI_ISL_8065615	EPI_ISL_8065709	EPI_ISL_8065818	EPI_ISL_8065911	EPI_ISL_11765137
EPI_ISL_1018098	EPI_ISL_2376389	EPI_ISL_8065616	EPI_ISL_8065711	EPI_ISL_8065815	EPI_ISL_8065913	EPI_ISL_11765148
EPI_ISL_1018071	EPI_ISL_8065535	EPI_ISL_8065617	EPI_ISL_8065716	EPI_ISL_8065808	EPI_ISL_8065914	EPI_ISL_11765164
EPI_ISL_1018074	EPI_ISL_2508377	EPI_ISL_8065618	EPI_ISL_8065718	EPI_ISL_8065811	EPI_ISL_8065916	EPI_ISL_11765141
EPI_ISL_1018073	EPI_ISL_2508378	EPI_ISL_8065619	EPI_ISL_8065703	EPI_ISL_8065812	EPI_ISL_8065917	EPI_ISL_11765146
EPI_ISL_1018072	EPI_ISL_2508374	EPI_ISL_8065582	EPI_ISL_8065712	EPI_ISL_8065814	EPI_ISL_8065919	EPI_ISL_11765135
EPI_ISL_1018075	EPI_ISL_2361920	EPI_ISL_8065586	EPI_ISL_8065717	EPI_ISL_8065816	EPI_ISL_8065918	EPI_ISL_11765136
EPI_ISL_1018080	EPI_ISL_2508379	EPI_ISL_8065593	EPI_ISL_8065721	EPI_ISL_8065819	EPI_ISL_8065920	EPI_ISL_11765139
EPI_ISL_1018076	EPI_ISL_2508385	EPI_ISL_8065620	EPI_ISL_8065719	EPI_ISL_8065820	EPI_ISL_8065922	EPI_ISL_11765158
EPI_ISL_1018079	EPI_ISL_2361921	EPI_ISL_8065621	EPI_ISL_8065720	EPI_ISL_8065821	EPI_ISL_8065925	EPI_ISL_11765163
EPI_ISL_1018077	EPI_ISL_2508376	EPI_ISL_8065622	EPI_ISL_8065724	EPI_ISL_8065822	EPI_ISL_8065923	EPI_ISL_11765165
EPI_ISL_1018078	EPI_ISL_2508375	EPI_ISL_8065623	EPI_ISL_8065722	EPI_ISL_8065824	EPI_ISL_8065924	EPI_ISL_11765169
EPI_ISL_1018081	EPI_ISL_2508372	EPI_ISL_8065628	EPI_ISL_8065723	EPI_ISL_8065810	EPI_ISL_8065928	EPI_ISL_11765172
EPI_ISL_1018083	EPI_ISL_2001079	EPI_ISL_8065629	EPI_ISL_8065729	EPI_ISL_8065826	EPI_ISL_8065926	EPI_ISL_11765147
EPI_ISL_1018084	EPI_ISL_2001077	EPI_ISL_8065631	EPI_ISL_8065736	EPI_ISL_8065825	EPI_ISL_8065927	EPI_ISL_11765190
EPI_ISL_1018082	EPI_ISL_2001078	EPI_ISL_8065641	EPI_ISL_8065738	EPI_ISL_8065827	EPI_ISL_8065929	EPI_ISL_11765179
EPI_ISL_1018087	EPI_ISL_2001070	EPI_ISL_8065645	EPI_ISL_8065741	EPI_ISL_8065828	EPI_ISL_8065931	EPI_ISL_11765244
EPI_ISL_1018085	EPI_ISL_2001074	EPI_ISL_8065639	EPI_ISL_8065727	EPI_ISL_8065830	EPI_ISL_8065932	EPI_ISL_11765250
EPI_ISL_1018086	EPI_ISL_2001075	EPI_ISL_8065625	EPI_ISL_8065728	EPI_ISL_8065831	EPI_ISL_8065930	EPI_ISL_11765259
EPI_ISL_2508370	EPI_ISL_2001076	EPI_ISL_8065624	EPI_ISL_8065735	EPI_ISL_8065829	EPI_ISL_8065933	EPI_ISL_11765263
EPI_ISL_2508371	EPI_ISL_2001073	EPI_ISL_8065626	EPI_ISL_8065733	EPI_ISL_8065832	EPI_ISL_8065934	EPI_ISL_11765252
EPI_ISL_2001081	EPI_ISL_2001069	EPI_ISL_8065627	EPI_ISL_8065725	EPI_ISL_8065834	EPI_ISL_8065935	EPI_ISL_11765243
EPI_ISL_2001082	EPI_ISL_2001071	EPI_ISL_8065630	EPI_ISL_8065726	EPI_ISL_8065835	EPI_ISL_8065936	EPI_ISL_11765246
EPI_ISL_2001083	EPI_ISL_2001072	EPI_ISL_8065632	EPI_ISL_8065731	EPI_ISL_8065836	EPI_ISL_8065937	EPI_ISL_11765189
EPI_ISL_2001084	EPI_ISL_8065536	EPI_ISL_8065633	EPI_ISL_8065732	EPI_ISL_8065837	EPI_ISL_8065938	EPI_ISL_11765195
EPI_ISL_2001085	EPI_ISL_8065538	EPI_ISL_8065634	EPI_ISL_8065737	EPI_ISL_8065838	EPI_ISL_8065940	EPI_ISL_11765245
EPI_ISL_2001086	EPI_ISL_8065537	EPI_ISL_8065635	EPI_ISL_8065739	EPI_ISL_8065839	EPI_ISL_8065939	EPI_ISL_11765197
EPI_ISL_2001087	EPI_ISL_8065539	EPI_ISL_8065638	EPI_ISL_8065742	EPI_ISL_8065840	EPI_ISL_8065941	EPI_ISL_11765266
EPI_ISL_2001088	EPI_ISL_8065540	EPI_ISL_8065642	EPI_ISL_8065743	EPI_ISL_8065841	EPI_ISL_8065943	EPI_ISL_11765279
EPI_ISL_2508387	EPI_ISL_8065541	EPI_ISL_8065643	EPI_ISL_8065730	EPI_ISL_8065850	EPI_ISL_8065942	EPI_ISL_11765274
EPI_ISL_2001080	EPI_ISL_8065544	EPI_ISL_8065644	EPI_ISL_8065734	EPI_ISL_8065843	EPI_ISL_8065944	EPI_ISL_11765283
EPI_ISL_2001089	EPI_ISL_8065545	EPI_ISL_8065646	EPI_ISL_8065740	EPI_ISL_8065849	EPI_ISL_8065945	EPI_ISL_11765275
EPI_ISL_2001090	EPI_ISL_8065542	EPI_ISL_8065636	EPI_ISL_8065746	EPI_ISL_8065842	EPI_ISL_8065946	EPI_ISL_11765261
EPI_ISL_2001091	EPI_ISL_8065543	EPI_ISL_8065637	EPI_ISL_8065753	EPI_ISL_8065848	EPI_ISL_8065947	EPI_ISL_11765271
EPI_ISL_2001098	EPI_ISL_8065546	EPI_ISL_8065640	EPI_ISL_8065744	EPI_ISL_8065851	EPI_ISL_8065948	EPI_ISL_11765257
EPI_ISL_2001096	EPI_ISL_8065547	EPI_ISL_8065647	EPI_ISL_8065751	EPI_ISL_8065844	EPI_ISL_8065949	EPI_ISL_11765194
EPI_ISL_2001097	EPI_ISL_8065548	EPI_ISL_8065648	EPI_ISL_8065755	EPI_ISL_8065845	EPI_ISL_8065950	EPI_ISL_11765186
EPI_ISL_2001092	EPI_ISL_8065550	EPI_ISL_8065649	EPI_ISL_8065754	EPI_ISL_8065846	EPI_ISL_8065951	EPI_ISL_11765181

gisaid_epi_isl	gisaid_epi_isl	gisaid_epi_isl	gisaid_epi_isl	gisaid_epi_isl	gisaid_epi_isl	gisaid_epi_isl
EPI_ISL_2001093	EPI_ISL_8065549	EPI_ISL_8065650	EPI_ISL_8065745	EPI_ISL_8065847	EPI_ISL_8065952	EPI_ISL_11765183
EPI_ISL_2001095	EPI_ISL_8065551	EPI_ISL_8065652	EPI_ISL_8065747	EPI_ISL_8065854	EPI_ISL_8065955	EPI_ISL_11765180
EPI_ISL_2001094	EPI_ISL_8065552	EPI_ISL_8065651	EPI_ISL_8065748	EPI_ISL_8065855	EPI_ISL_8065954	EPI_ISL_11765185
EPI_ISL_2508388	EPI_ISL_8065553	EPI_ISL_8065653	EPI_ISL_8065749	EPI_ISL_8065852	EPI_ISL_8065953	EPI_ISL_11765178
EPI_ISL_11765220	EPI_ISL_11765344	EPI_ISL_11765209	EPI_ISL_11765390	EPI_ISL_11765352	EPI_ISL_11765395	EPI_ISL_11765254
EPI_ISL_11765204	EPI_ISL_11765405	EPI_ISL_11765223	EPI_ISL_11765385	EPI_ISL_11765371	EPI_ISL_11765369	EPI_ISL_11765270
EPI_ISL_11765210	EPI_ISL_11765416	EPI_ISL_11765211	EPI_ISL_11765375	EPI_ISL_11765406	EPI_ISL_11765364	EPI_ISL_11765255
EPI_ISL_11765212	EPI_ISL_11765417	EPI_ISL_11765222	EPI_ISL_11765386	EPI_ISL_11765409	EPI_ISL_11765393	EPI_ISL_11765282
EPI_ISL_11765215	EPI_ISL_11765418	EPI_ISL_11765288	EPI_ISL_11765339	EPI_ISL_11765341	EPI_ISL_11765387	EPI_ISL_11765264
EPI_ISL_11765199	EPI_ISL_11765419	EPI_ISL_11765218	EPI_ISL_11765349	EPI_ISL_11765336	EPI_ISL_11765384	EPI_ISL_11765187
EPI_ISL_11765201	EPI_ISL_11765420	EPI_ISL_11765337	EPI_ISL_11765363	EPI_ISL_11765381	EPI_ISL_11765392	EPI_ISL_11765278
EPI_ISL_11765237	EPI_ISL_11765414	EPI_ISL_11765365	EPI_ISL_11765366	EPI_ISL_11765377	EPI_ISL_11765389	EPI_ISL_11765273
EPI_ISL_11765216	EPI_ISL_11765421	EPI_ISL_11765362	EPI_ISL_11765350	EPI_ISL_11765367	EPI_ISL_11765394	EPI_ISL_11765251
EPI_ISL_11765200	EPI_ISL_11765422	EPI_ISL_11765342	EPI_ISL_11765340	EPI_ISL_11765397	EPI_ISL_11765374	EPI_ISL_11765184
EPI_ISL_11765205	EPI_ISL_11765423	EPI_ISL_11765356	EPI_ISL_11765359	EPI_ISL_11765347	EPI_ISL_11765353	EPI_ISL_11765262
EPI_ISL_11765219	EPI_ISL_11765424	EPI_ISL_11765335	EPI_ISL_11765376	EPI_ISL_11765343	EPI_ISL_11765398	EPI_ISL_11765258
EPI_ISL_11765236	EPI_ISL_11765425	EPI_ISL_11765345	EPI_ISL_11765383	EPI_ISL_11765357	EPI_ISL_11765399	EPI_ISL_11765192
EPI_ISL_11765289	EPI_ISL_11765426	EPI_ISL_11765388	EPI_ISL_11765396	EPI_ISL_11765372	EPI_ISL_11765378	EPI_ISL_11765247
EPI_ISL_11765214	EPI_ISL_11765427	EPI_ISL_11765361	EPI_ISL_11765334	EPI_ISL_11765413	EPI_ISL_11765391	EPI_ISL_11765260
EPI_ISL_11765203	EPI_ISL_11765415	EPI_ISL_11765354	EPI_ISL_11765358	EPI_ISL_11765404	EPI_ISL_11765382	EPI_ISL_11765248
EPI_ISL_11765198	EPI_ISL_11765408	EPI_ISL_11765346	EPI_ISL_11765379	EPI_ISL_11765411	EPI_ISL_11765280	EPI_ISL_11765256
EPI_ISL_11765213	EPI_ISL_11765407	EPI_ISL_11765355	EPI_ISL_11765373	EPI_ISL_11765412	EPI_ISL_11765277	EPI_ISL_11765191
EPI_ISL_11765242	EPI_ISL_11765400	EPI_ISL_11765380	EPI_ISL_11765338	EPI_ISL_11765402	EPI_ISL_11765269	EPI_ISL_11765188
EPI_ISL_11765221	EPI_ISL_11765401	EPI_ISL_11765348	EPI_ISL_11765351	EPI_ISL_11765403	EPI_ISL_11765284	EPI_ISL_11765253
EPI_ISL_11765235	EPI_ISL_11765410	EPI_ISL_11765368	EPI_ISL_11765370	EPI_ISL_11765360	EPI_ISL_11765268	EPI_ISL_11765193
EPI_ISL_11765249	EPI_ISL_11765196	EPI_ISL_11765324	EPI_ISL_11765326	EPI_ISL_11765318	EPI_ISL_11765299	EPI_ISL_11765295
EPI_ISL_11765182	EPI_ISL_8065986	EPI_ISL_11765311	EPI_ISL_11765310	EPI_ISL_11765285	EPI_ISL_11765319	EPI_ISL_11765330
EPI_ISL_11765265	EPI_ISL_11765293	EPI_ISL_11765320	EPI_ISL_11765294	EPI_ISL_11765329	EPI_ISL_11765315	EPI_ISL_11765332
EPI_ISL_11765276	EPI_ISL_11765321	EPI_ISL_11765328	EPI_ISL_11765307	EPI_ISL_11765304	EPI_ISL_11765316	EPI_ISL_11765325
EPI_ISL_11765281	EPI_ISL_11765322	EPI_ISL_11765290	EPI_ISL_11765312	EPI_ISL_11765306	EPI_ISL_11765323	EPI_ISL_11765298
EPI_ISL_11765272	EPI_ISL_11765233	EPI_ISL_11765309	EPI_ISL_11765287	EPI_ISL_11765303	EPI_ISL_11765302	EPI_ISL_11765308
EPI_ISL_11765267	EPI_ISL_11765229	EPI_ISL_11765301	EPI_ISL_11765296	EPI_ISL_11765300	EPI_ISL_11765331	EPI_ISL_11765313
EPI_ISL_11765305	EPI_ISL_11765291	EPI_ISL_11765230	EPI_ISL_11765225	EPI_ISL_11765241	EPI_ISL_11765224	EPI_ISL_11765238
EPI_ISL_11765333	EPI_ISL_11765292	EPI_ISL_11765239	EPI_ISL_11765240	EPI_ISL_11765207	EPI_ISL_11765228	
EPI_ISL_11765286	EPI_ISL_11765314	EPI_ISL_11765231	EPI_ISL_11765202	EPI_ISL_11765208	EPI_ISL_11765217	
EPI_ISL_11765327	EPI_ISL_11765297	EPI_ISL_11765226	EPI_ISL_11765206	EPI_ISL_11765234	EPI_ISL_11765227	
Samples from returning International travelers						
EPI_ISL_422398	EPI_ISL_422400	EPI_ISL_5659351	EPI_ISL_5659352	EPI_ISL_5659353	EPI_ISL_5659354	EPI_ISL_5659355
EPI_ISL_5659356	EPI_ISL_5659369	EPI_ISL_4253817	EPI_ISL_5736566	EPI_ISL_5736546	EPI_ISL_5736544	EPI_ISL_4253803
EPI_ISL_5659357	EPI_ISL_5659370	EPI_ISL_4253816	EPI_ISL_5736578	EPI_ISL_5736552	EPI_ISL_5736547	EPI_ISL_4253802
EPI_ISL_5659358	EPI_ISL_5659371	EPI_ISL_4253815	EPI_ISL_5736556	EPI_ISL_5736571	EPI_ISL_5736575	EPI_ISL_4253801
EPI_ISL_5659359	EPI_ISL_4253826	EPI_ISL_4253814	EPI_ISL_5736579	EPI_ISL_5736572	EPI_ISL_5736573	EPI_ISL_4253800
EPI_ISL_5659361	EPI_ISL_4253825	EPI_ISL_4253813	EPI_ISL_5736557	EPI_ISL_5736561	EPI_ISL_4253811	EPI_ISL_4253799
EPI_ISL_5659362	EPI_ISL_4253824	EPI_ISL_4253812	EPI_ISL_5736545	EPI_ISL_5736539	EPI_ISL_4253810	EPI_ISL_4253798
EPI_ISL_5659363	EPI_ISL_4253823	EPI_ISL_5736570	EPI_ISL_5736543	EPI_ISL_5736567	EPI_ISL_4253809	EPI_ISL_4253797
EPI_ISL_5659364	EPI_ISL_4253822	EPI_ISL_5736553	EPI_ISL_5736538	EPI_ISL_5736568	EPI_ISL_4253808	EPI_ISL_4253796
EPI_ISL_5659365	EPI_ISL_4253821	EPI_ISL_5736554	EPI_ISL_5736558	EPI_ISL_5736533	EPI_ISL_4253807	EPI_ISL_4253795
EPI_ISL_5659366	EPI_ISL_4253820	EPI_ISL_5736555	EPI_ISL_5736576	EPI_ISL_5736537	EPI_ISL_4253806	EPI_ISL_4253794
EPI_ISL_5659367	EPI_ISL_4253819	EPI_ISL_5736536	EPI_ISL_5736559	EPI_ISL_5736532	EPI_ISL_4253805	EPI_ISL_4253793

gisaid_epi_isl	gisaid_epi_isl	gisaid_epi_isl	gisaid_epi_isl	gisaid_epi_isl	gisaid_epi_isl	gisaid_epi_isl
EPI_ISL_5659368	EPI_ISL_4253818	EPI_ISL_5736565	EPI_ISL_5736560	EPI_ISL_5736577	EPI_ISL_4253804	EPI_ISL_4253792
EPI_ISL_6939052	EPI_ISL_6939063	EPI_ISL_6939022	EPI_ISL_6944053	EPI_ISL_6944035	EPI_ISL_6944018	EPI_ISL_4253791
EPI_ISL_6939042	EPI_ISL_6939047	EPI_ISL_6939023	EPI_ISL_6944054	EPI_ISL_6944036	EPI_ISL_6944017	EPI_ISL_4253790
EPI_ISL_6939043	EPI_ISL_6939041	EPI_ISL_6939024	EPI_ISL_6944055	EPI_ISL_6944037	EPI_ISL_6944019	EPI_ISL_4919701
EPI_ISL_6939044	EPI_ISL_6939051	EPI_ISL_6939066	EPI_ISL_6944056	EPI_ISL_6944038	EPI_ISL_6944020	EPI_ISL_4919706
EPI_ISL_6939035	EPI_ISL_6939068	EPI_ISL_6939055	EPI_ISL_6944057	EPI_ISL_6944039	EPI_ISL_6944021	EPI_ISL_4919708
EPI_ISL_6939050	EPI_ISL_6939064	EPI_ISL_6939025	EPI_ISL_6944058	EPI_ISL_6944040	EPI_ISL_6944026	EPI_ISL_4919709
EPI_ISL_6939045	EPI_ISL_6939038	EPI_ISL_6939026	EPI_ISL_6944059	EPI_ISL_6944041	EPI_ISL_6944022	EPI_ISL_4919711
EPI_ISL_2873840	EPI_ISL_6939039	EPI_ISL_6939027	EPI_ISL_6944060	EPI_ISL_6944042	EPI_ISL_6944023	EPI_ISL_4919699
EPI_ISL_2873843	EPI_ISL_6939057	EPI_ISL_6939028	EPI_ISL_6944061	EPI_ISL_6944043	EPI_ISL_6944024	EPI_ISL_4919702
EPI_ISL_2873844	EPI_ISL_6939033	EPI_ISL_6939059	EPI_ISL_6944062	EPI_ISL_6944044	EPI_ISL_6944025	EPI_ISL_4919704
EPI_ISL_5751221	EPI_ISL_6939049	EPI_ISL_6939060	EPI_ISL_6939017	EPI_ISL_6944046	EPI_ISL_6944028	EPI_ISL_6944009
EPI_ISL_5751201	EPI_ISL_6939067	EPI_ISL_6939058	EPI_ISL_6939069	EPI_ISL_6944045	EPI_ISL_6944027	EPI_ISL_6944010
EPI_ISL_5751176	EPI_ISL_6939030	EPI_ISL_6939029	EPI_ISL_6939018	EPI_ISL_6944047	EPI_ISL_6944029	EPI_ISL_6944011
EPI_ISL_5751178	EPI_ISL_6939034	EPI_ISL_6939048	EPI_ISL_6939019	EPI_ISL_6944048	EPI_ISL_6944030	EPI_ISL_6944013
EPI_ISL_2873846	EPI_ISL_6939031	EPI_ISL_6939032	EPI_ISL_6939037	EPI_ISL_6944049	EPI_ISL_6944031	EPI_ISL_6944012
EPI_ISL_2873847	EPI_ISL_6939056	EPI_ISL_6939053	EPI_ISL_6939020	EPI_ISL_6944050	EPI_ISL_6944032	EPI_ISL_6944014
EPI_ISL_5751057	EPI_ISL_6939054	EPI_ISL_6939061	EPI_ISL_6939021	EPI_ISL_6944051	EPI_ISL_6944033	EPI_ISL_6944015
EPI_ISL_5751228	EPI_ISL_6939065	EPI_ISL_6939062	EPI_ISL_6939040	EPI_ISL_6944052	EPI_ISL_6944034	EPI_ISL_6944016
EPI_ISL_5751078	EPI_ISL_4602064	EPI_ISL_2873862	EPI_ISL_5751218	EPI_ISL_5334612	EPI_ISL_5751094	EPI_ISL_4602053
EPI_ISL_2873848	EPI_ISL_5751193	EPI_ISL_5751071	EPI_ISL_5751179	EPI_ISL_5334611	EPI_ISL_4602040	EPI_ISL_4602054
EPI_ISL_2873849	EPI_ISL_2873857	EPI_ISL_2873863	EPI_ISL_5751107	EPI_ISL_5334610	EPI_ISL_4602041	EPI_ISL_4602055
EPI_ISL_2873850	EPI_ISL_5751142	EPI_ISL_2873864	EPI_ISL_4602070	EPI_ISL_5751121	EPI_ISL_4602042	EPI_ISL_4602060
EPI_ISL_2873851	EPI_ISL_5751161	EPI_ISL_4602067	EPI_ISL_2873868	EPI_ISL_5334609	EPI_ISL_4602043	EPI_ISL_4602056
EPI_ISL_2873852	EPI_ISL_2873858	EPI_ISL_5751210	EPI_ISL_2873869	EPI_ISL_5751215	EPI_ISL_4602044	EPI_ISL_4602057
EPI_ISL_2873853	EPI_ISL_2873859	EPI_ISL_2873865	EPI_ISL_2873870	EPI_ISL_5334608	EPI_ISL_4602045	EPI_ISL_4602058
EPI_ISL_5751127	EPI_ISL_2873860	EPI_ISL_4602069	EPI_ISL_2873871	EPI_ISL_5751084	EPI_ISL_4602046	EPI_ISL_4602059
EPI_ISL_2873854	EPI_ISL_5751136	EPI_ISL_5751202	EPI_ISL_2873872	EPI_ISL_5751100	EPI_ISL_4602047	EPI_ISL_4602061
EPI_ISL_2873855	EPI_ISL_5751186	EPI_ISL_2873866	EPI_ISL_2873873	EPI_ISL_5751097	EPI_ISL_4602048	EPI_ISL_5334607
EPI_ISL_5751204	EPI_ISL_4602065	EPI_ISL_2873867	EPI_ISL_5751061	EPI_ISL_5751083	EPI_ISL_4602049	EPI_ISL_5334606
EPI_ISL_2873856	EPI_ISL_4602066	EPI_ISL_5751188	EPI_ISL_5334615	EPI_ISL_5751099	EPI_ISL_4602050	EPI_ISL_5334605
EPI_ISL_4602062	EPI_ISL_5751151	EPI_ISL_5751152	EPI_ISL_5334614	EPI_ISL_5751093	EPI_ISL_4602051	EPI_ISL_5334604
EPI_ISL_4602063	EPI_ISL_2873861	EPI_ISL_5751066	EPI_ISL_5334613	EPI_ISL_5751170	EPI_ISL_4602052	EPI_ISL_5334603
EPI_ISL_5334602	EPI_ISL_5334601	EPI_ISL_5334600	EPI_ISL_5334599	EPI_ISL_5334598	EPI_ISL_5334597	EPI_ISL_5334596
EPI_ISL_5334595	EPI_ISL_5334594	EPI_ISL_5334593	EPI_ISL_5659350	EPI_ISL_422382	EPI_ISL_422404	EPI_ISL_422384

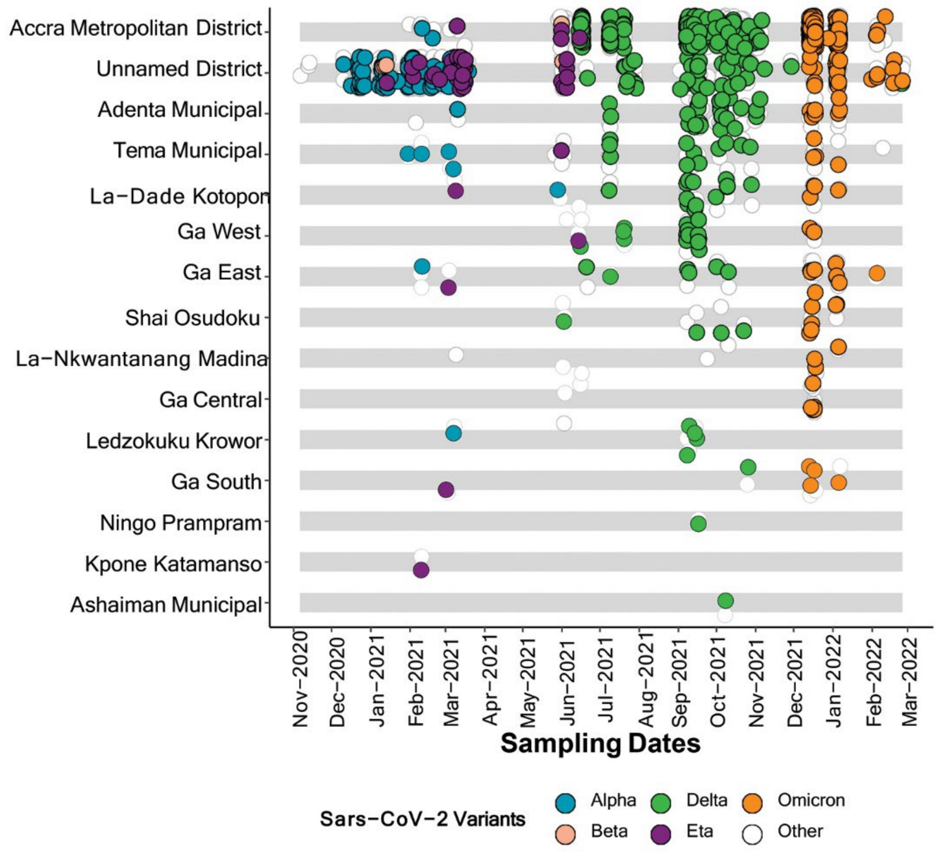

Appendix Figure 1. Overview of SARS-CoV-2 lineages over time by districts. Samples that came from within the Greater Accra Region but without a clear indication of the specific district have their districts indicated as ‘Unnamed District’.

A

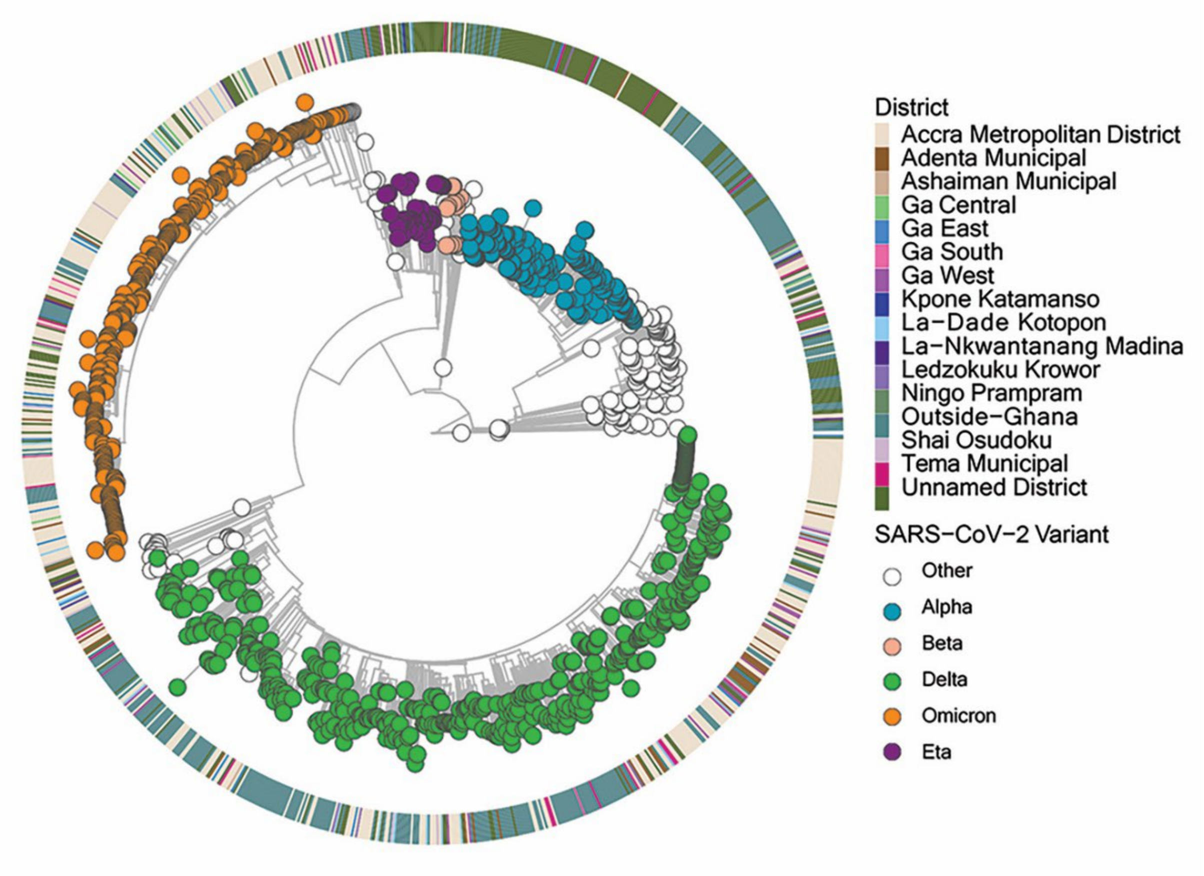

District
Accra Metropolitan District
Adenta Municipal
Ashaiman Municipal
Ga Central
Ga East
Ga South
Ga West
Kpone Katamanso
La−Dade Kotopon
La−Nkwantanang Madina
Ledzokuku Krowor
Ningo Prampram
Outside−Ghana
Shai Osudoku
Tema Municipal
Unnamed District
SARS−CoV−2 Variant
Other
Alpha
Beta
Delta
Omicron
Eta

B

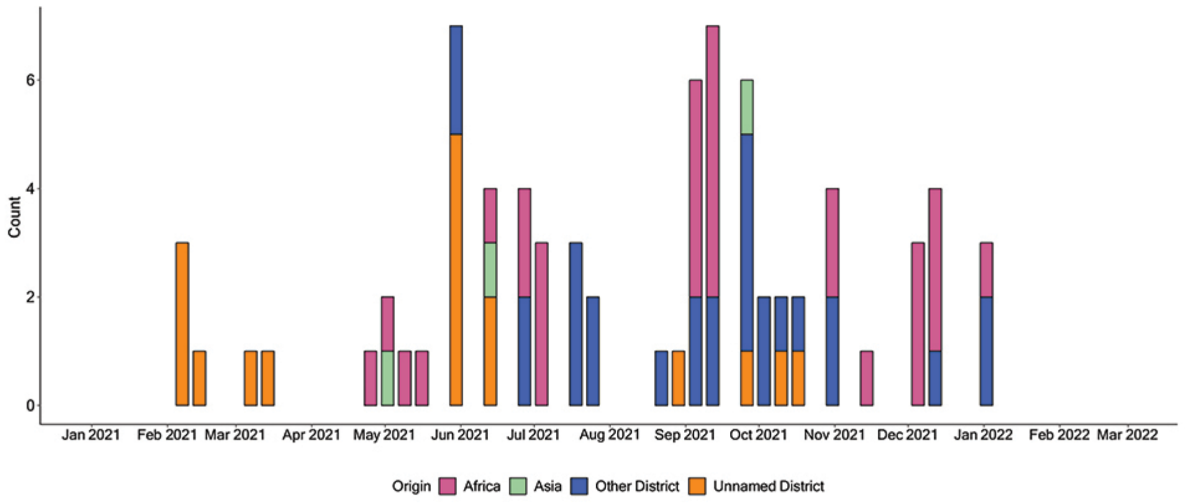

Count
0
2
4
6
Jan 2021
Feb 2021
Mar 2021
Apr 2021
May 2021
Jun 2021
Jul 2021
Aug 2021
Sep 2021
Oct 2021
Nov 2021
Dec 2021
Jan 2022
Feb 2022
Mar 2022
Origin
Africa
Asia
Other District
Unnamed District

C

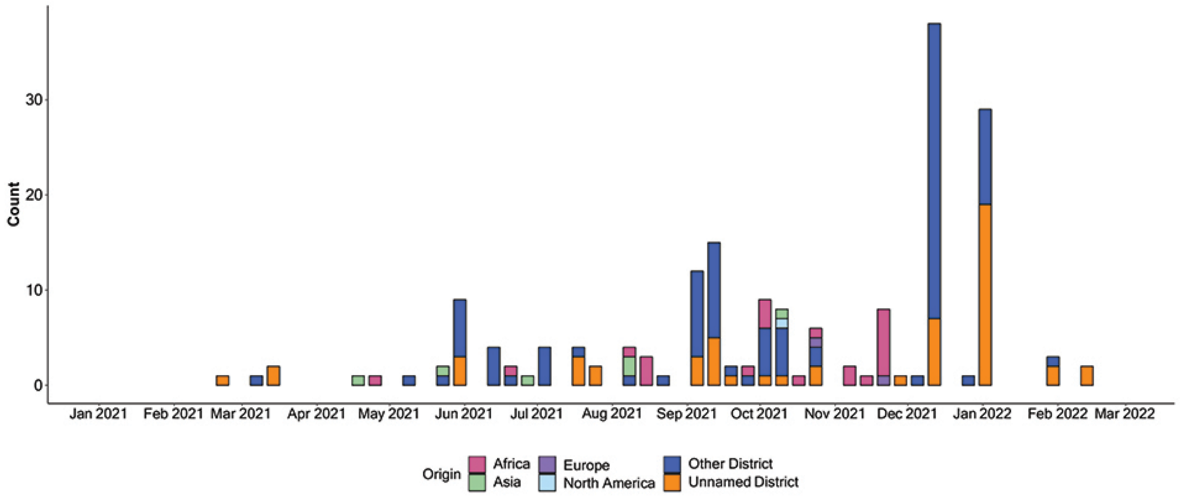

D

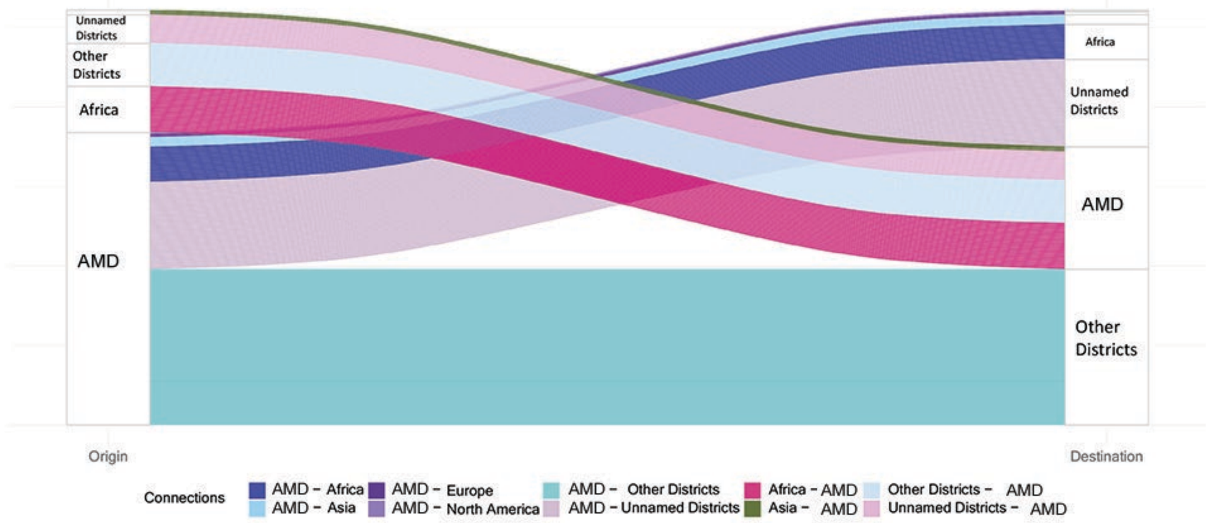

Appendix Figure 2. Phylogenetic relationship and spread of SARS-CoV-2 lineages in the Greater-Accra region (GAR) of Ghana. A) Rooted maximum-likelihood tree of SARS-CoV-2 variants in the GAR inferred from whole-genome sequencing data. The colors of the tips represent the SARS-CoV-2 variants showing the 5 major variants recorded; all others were classified as “Other.” The colors of the heatmap indicate the district in the GAR from which the sample was collected. Samples from returning international travelers who tested positive for COVID-19 at arrival at the Kotoka International Airport (Accra, Ghana)

are classified as “Outside Ghana.” The tree was rooted at the Wuhan reference genome (GenBank accession no. NC_045512.2). B) SARS-CoV-2 importation events showing the number of events into the Accra Metropolitan District (AMD). C) SARS-CoV-2 exportation events showing the number of events from the AMD to other districts and regions. D) Alluvial plot showing the flow of SARS-CoV-2 importation and exportation between the regions. For panels B, C and D, 2020 data were excluded from the analysis because of paucity. The “Other District” category is combination of all the named districts excluding the AMD. For all panels, samples that came from within the GAR but without a clear indication of the specific district are indicated as “Unnamed District.”
